# A review on the occurrence of companion vector-borne diseases in pet animals in Latin America

**DOI:** 10.1186/s13071-019-3407-x

**Published:** 2019-03-28

**Authors:** Ricardo G. Maggi, Friederike Krämer

**Affiliations:** 1 Department of Clinical Sciences and the Intracellular Pathogens Research Laboratory, College of Veterinary Medicine, North Carolina State University, Raleigh, NC USA; 2 Institute of Parasitology, Faculty of Veterinary Medicine, Leipzig University, Leipzig, Germany

**Keywords:** Companion vector-borne diseases (CVBDs), Dog, Cat, Occurrence, Vector, Latin America (LATAM), Prevalence

## Abstract

Companion vector-borne diseases (CVBDs) are an important threat for pet life, but may also have an impact on human health, due to their often zoonotic character. The importance and awareness of CVBDs continuously increased during the last years. However, information on their occurrence is often limited in several parts of the world, which are often especially affected. Latin America (LATAM), a region with large biodiversity, is one of these regions, where information on CVBDs for pet owners, veterinarians, medical doctors and health workers is often obsolete, limited or non-existent. In the present review, a comprehensive literature search for CVBDs in companion animals (dogs and cats) was performed for several countries in Central America (Belize, Caribbean Islands, Costa Rica, Cuba, Dominican Republic, El Salvador, Guatemala, Honduras, Mexico, Nicaragua, Panama, Puerto Rico) as well as in South America (Argentina, Bolivia, Brazil, Chile, Colombia, Ecuador, French Guiana, Guyana (British Guyana), Paraguay, Peru, Suriname, Uruguay, Venezuela) regarding the occurrence of the following parasitic and bacterial diseases: babesiosis, heartworm disease, subcutaneous dirofilariosis, hepatozoonosis, leishmaniosis, trypanosomosis, anaplasmosis, bartonellosis, borreliosis, ehrlichiosis, mycoplasmosis and rickettsiosis. An overview on the specific diseases, followed by a short summary on their occurrence per country is given. Additionally, a tabular listing on positive or non-reported occurrence is presented. None of the countries is completely free from CVBDs. The data presented in the review confirm a wide distribution of the CVBDs in focus in LATAM. This wide occurrence and the fact that most of the CVBDs can have a quite severe clinical outcome and their diagnostic as well as therapeutic options in the region are often difficult to access and to afford, demands a strong call for the prevention of pathogen transmission by the use of ectoparasiticidal and anti-feeding products as well as by performing behavioural changes.

## Background

Companion vector-borne diseases (CVBDs) have among others a major impact on the welfare of pets. They may also represent a constant risk to humans due to their zoonotic nature, which emphasizes the importance of pets as reservoirs.

In Latin America (LATAM), a region with one of the largest biodiversities in the world, a combination of factors such as intensification of agricultural practices, landscape modification, poor ecosystem protection and potentially slight unstable economics, creates host populations conducive to the performance and persistence of parasites and vectors.

This is especially important for CVBDs affecting dogs and cats as companion animals, as a significant proportion of those (i.e. 52–75%) [1, 2], even though owned by pet holders, roam freely, besides an exploding number of stray dogs and cats. In LATAM, the lack of sensitive awareness of animal welfare and disease issues, the restricted economic and technological access to proper veterinary care, and the absence of responsible pet ownership, have created conditions for the emergence and persistence of many diseases that ultimately will affect people, livestock, and wildlife [3–10]. Besides, socio-economic, demographic and ecological factors, including globalization, increase in international trade, tourism and travel, climate change and its effect on vector distribution in time and space, have also to be reconsidered.

This article summarizes the data of reported detection (or prevalence when available) of the most significant CVBDs affecting companion animals in LATAM in tabular form and as detailed information per country and discusses research gaps to be addressed in future studies. In case of very scarce published data, additionally the occurrence of the pathogens in potential vectors, wild canids or felids and in humans is listed, to illustrate the fact that the pathogen is occurring in a respective region, even though not officially reported in companion animals so far. Beforehand a brief introduction on the diseases, usually followed by a short summary or references for more detailed data on diagnostic methods, treatment indications and ways of prevention are given.

Generally, for many of the vector-borne diseases (VBDs) described here, diverse diagnostic tests are available (microscopic, serological, molecular). Nevertheless, besides their different performance regarding sensitivity and specificity in acute and chronic disease, only few are readily available as diagnostic tools at most clinical practices in the reported LATAM regions.

## Parasitic diseases

### Babesiosis

Babesiosis in pet animals in LATAM is mainly caused by *Babesia vogeli* and *Babesia gibsoni* [11–13]. The disease has been reported in many areas especially of South America, whereas reports from Central America are scarce so far. *Babesia vogeli* is transmitted directly *via* tick bites [*Rhipicephalus sanguineus* (*sensu lato*)], whereas *B. gibsoni* in LATAM is expected to be transmitted *via* blood transfer through dog bites, blood transfusions and transplacental supply [14–18]. Clinical signs, depending on the species, and further details on clinical and laboratory findings can be found in Irwin [14]. As diagnosis microscopy remains the simplest and most accessible diagnostic test. Different sensitivity during the cause of disease may be supported by molecular methods (see Irwin [14] for details). Treatment does not eliminate the parasite, but only reduces parasitemia and supports resolution of clinical signs and is summarized elsewhere [14]. Animals diagnosed with *Babesia* spp. should be considered permanent carriers of the infection. Due to the missing elimination of the pathogen during treatment, vaccines have been introduced with variable efficacy (see Irwin [14] for summary). According to the authors’ knowledge, the vaccines are only available in Europe, so that prevention of vector exposure in form of acaricidal treatment is essential especially for LATAM.

### Dirofilariosis

Dirofilariosis is caused by *Dirofilaria immitis*, presenting as an important disease, causing cardiopulmonary problems and even death in dogs worldwide and commonly known as canine heartworm disease, and by *Dirofilaria repens*, a subcutaneous parasite of dogs and cats in Europe, Africa and Asia.

#### Canine heartworm disease

Canine heartworm disease has a wide distribution in LATAM (except Belize, Guatemala, Panama, French Guiana, Chile and Uruguay; for specific data see individual country sections). The pathogen is transmitted by several mosquito species. As a mosquito-transmitted disease, it is more prevalent in tropical and subtropical regions, due to favorable conditions for mosquito propagation [19–21]. Clinical signs vary from nearly asymptomatic to very severe and are listed elsewhere [22–24]. Diagnostic methods include microfilaria testing of blood samples, ideally after a concentration technique (modified Knottʼs test or filtration test), and antigen testing. For details on different test sensitivities and combinations please see the guidelines of the American Heartworm Society (AHS) [25]. Treatment against heartworm varies depending on the severity of the disease and always aims to improve the clinical condition and to eliminate all life stages of the heartworms with minimal post-treatment complications. Prevention by the use of chemoprophylactic drugs is strongly recommended year-round in endemic areas. For full recommendations see the guidelines of the Tropical Council of Companion Animal Parasites (TroCCAP) [26] and the AHS [25]. Prevention of vector exposure on the basis of antifeeding and/or insecticidal treatments and by the use of mosquito screens etc. and reduction of suitable breeding sites for mosquitoes support a successful prevention scheme.

#### Subcutaneous dirofilariosis

Subcutaneous dirofilariosis is a filarial disease caused by *D. repens*. Again, transmitted by diverse mosquito species, adult worms are located mainly in subcutaneous tissues. The presence of adult *D. repens* worms in subcutaneous tissues and/or subcutaneous nodules [27] often goes unnoticed but can also cause cutaneous disorders [28–31], as well as extradermic symptoms [32]. For further details on the parasite see also Genchi et al. [33] and Simón et al. [34]. The disease is mainly distributed in Europe, Africa and Asia, and only single reports with closely related variants for LATAM exist [35, 36]. Diagnostic methods usually rely on the detection of microfilariae in blood samples as described for *D. immitis*. If clinically apparent, surgical excision and subsequent histopathological confirmation is the general treatment option. From the medical standpoint, here especially regarding the Old World, *D. repens* is the most frequent and most widely distributed in comparison to *D. immitis* and other *Dirofilaria* species [37] and thus especially of zoonotic importance. For the New World, different species might be involved.

### Hepatozoonosis

Hepatozoonosis has been described infrequently in LATAM, despite high prevalences reported from some rural areas of Brazil and Costa Rica [38–41]. Canine hepatozoonosis is caused by *Hepatozoon canis*, a protozoan transmitted by ingestion of ticks containing mature *H. canis* oocysts. Clinical signs of hepatozoonosis and laboratory changes can be found in Sherding [42] and Baneth [43]. The disease is debilitating and often fatal if not treated. *Hepatozoon canis* infection is frequently diagnosed by microscopic detection of intracellular gamonts in stained blood smears. Antibody detection and molecular detection *via* PCR are also available; see Baneth [43] for further details. Complete elimination may frequently not be achievable [44]; for details on treatment see Baneth [43]. Prognosis of treated dogs depends on the parasitaemia. Prevention of vector exposure in form of ectoparasiticidal treatment is supporting the protection against *H. canis*.

### Leishmaniosis

Leishmaniosis in LATAM is mainly caused by *Leishmania infantum* (syn. *Leishmania chagasi*). Other species (e.g. *Leishmania braziliensis*, *Leishmania amazonensis*) can also be involved in causing disease. While *L. infantum* is the most important causative agent of canine visceral leishmaniosis in South America [45], *L. amazonensis* has as well been reported causing visceral leishmaniosis in dogs [46], whereas *L. braziliensis* has been detected in dogs with cutaneous leishmaniosis [47]. The parasites are transmitted mainly by sand flies (for LATAM, species of the genus *Lutzomyia* [48, 49]). Clinical signs can vary from very subtle (asymptomatic) to very severe. Clinical staging has been deeply elaborated by LeishVet and published in Solano-Gallego et al. [50, 51] for dogs and in Pennisi et al. [52] for cats. The most useful diagnostic approaches include demonstration of the parasite DNA in blood or other tissues and detection of specific serum anti-leishmanial antibodies [50, 51, 53–55], but might not be available in all regions in LATAM. Direct parasite detection by cytology and further diagnostic approaches are described and evaluated in the LeishVet guidelines for the practical management of canine leishmaniosis [51]. Treatment for leishmaniosis is controversial in many countries and includes several anti-leishmanial drugs. Treatment regimens for the different stages of disease have been published in Solano-Gallego et al. [50, 56]. In South America, canine leishmaniosis treatment might often not routinely be performed. The elimination of seropositive dogs (euthanasia/culling program) has been practiced, e.g. in Brazil, even though for Brazil this control measure has been subject of intense, ongoing debate, due to ethical reasons and the lack of scientific evidence supporting the effectiveness of this strategy [57–59]. Meanwhile, a veterinary drug based on oral miltefosine has been authorized for marketing in Brazil [60]. As *L. infantum* has zoonotic potential, and dogs are regarded as the main reservoir for this pathogen, prevention is essential from the standpoint of animal welfare as well as under the aspect of One Health. Besides a reduced exposure to sand flies based on behavioral codes, insecticidal prophylaxis is strongly recommended. Another approach to help controlling canine leishmaniosis was the introduction of a vaccine, which has been licensed in Brazil in 2014 and which proved to be effective to reduce the number of canine visceral leishmaniosis cases in vaccinated animals [61].

### Trypanosomosis

Trypanosomosis is a disease of human medical and veterinary importance caused mainly by *Trypanosoma cruzi*. This disease, also known as Chagas disease or American trypanosomosis, has been recognized by the World Health Organization (WHO) as the most important parasitic disease in the Americas by disability adjusted life years (DALYs) [62]. An estimated 99.8% of the disease burden occurs in LATAM and the Caribbean region [63–67]. Dogs are considered the predominant domestic reservoir for Chagas disease (*T. cruzi*) in many areas of endemicity [68]. Other trypanosomatid pathogen species such as *Trypanosma evansi* and *Trypanosoma rangeli* have been also implicated in infections in dogs. The recognized vectors for *T. cruzi* are triatomine species, while *T. evansi* is transmitted in several ways *via* biting insects, sucking insects and vampire bats [69, 70]. Clinical signs of *T. cruzi* infection in dogs may vary from acute to chronic disease [71]. Regarding *T. evansi*, dogs usually experience acute fatal infections [72, 73]. The most common and easiest diagnostic method for *Trypanosoma* infection is microscopic identification in a blood smear or the buffy coat, successful during the acute stage. For chronic Chagas disease, diagnosis relies on serological tests. Recommendations on serological tests in the chronic phase [74–81] and a detailed review [82] offer further information. Regarding dogs, there are few studies focusing on the diagnosis of *T. cruzi* infection [83–87]and even fewer in naturally infected dogs using recombinant antigens [88]. Different antigens have been tested by Brasil et al. [82] for their suitability in dogs. The drug of choice for treatment is benznidazole, but nifurtimox can also be used [89]. Symptomatic treatment for heart failure and arrhythmias is also recommended [90]. Prevention of disease transmission especially in humans is among others heavily relying in vector control [68]. As the dog is a major reservoir for human Chagas disease, vector control should also include the prevention of disease transmission in dogs.

## Bacterial diseases

### Anaplasmosis

Anaplasmosis in dogs and cats can be caused by *Anaplasma phagocytophilum*, causative agent of canine granulocytic anaplasmosis (CGA), mainly occurring in temperate zones of the world, and *Anaplasma platys*, the pathogenic agent of canine cyclic thrombocytopenia, occurring worldwide with a higher incidence in tropical and and subtropical areas [91]. For LATAM, both species have been reported in infections, but mainly with *A. platys*.

Even though most dogs naturally infected with *A. phagocytophilum* probably remain healthy, clinical signs [92–95] and hematological changes [94] have been reported. In general, infection with *A. platys* may go along subclinically (e.g. in the USA and Australia), but distinct clinical abnormalities have also been reported, besides hematological abnormalities (in Europe and Israel [96, 97]). A good overview for both pathogens is given in Sainz et al. [98]. In the majority of dogs both types of anaplasmoses pose a diagnostic challenge and clinical and hematological abnormalities should be combined with laboratory and diagnostic tests. Microscopic detection of morulae (intracytoplasmatic inclusions) in neutrophils (for *A. phagocytophilum*) or platelets (for *A. platys*) in stained blood smears is indicative for an infection with an intracytoplasmic coccus, but not distinguishing between *A. phagocytophilum* and other *Ehrlichia* spp. [98], respectively sensitivity appears to be rather low for *A. platys* [99], so that serology and ideally PCR should also be performed additionally for definitive diagnosis. For details on diagnostic interpretation see Sainz et al. [98] and Carrade et al. [100]. For treatment of both pathogen infections doxycycline is effective (see Sainz et al. [98] for a summary on treatment parameters). The prevention of anaplasmosis in dogs must be focused on tick control, even though the vector of *A. platys* is still unknown or unproven. But ticks of various genera (e.g. *Rhipicephalus*, *Dermacentor* and *Ixodes*) have been found naturally infected by *A. platys* around the world [101–105]. Regarding *A. phagocytophilum*, tick control is an essential demand enforced even by the zoonotic character of the pathogen.

### Bartonellosis

Bartonellosis has been described in dogs and cats sporadically in LATAM. The most common species detected in dogs are *Bartonella henselae* and *Bartonella vinsonii berkhoffii*, while *B. henselae* and *Bartonella clarridgeiae* are the most commonly detected species in cats [106]. *Bartonella* species can be transmitted to companion animals and humans by several insects, including fleas, sand flies, lice, bed bugs, mites and ticks (e.g. [107–131]), and also directly by cat scratches, bites, blood transfusion and organ transplant (even though the last two have been mostly reported in humans) (e.g. [130, 132–150]. Clinical appearance may include a large variety of signs (e.g. [143, 144, 151–170] and laboratory abnormalities [165, 167, 171–173]. Diagnosis of *Bartonella* infection can be performed by IFA test, PCR, or blood culture. Unfortunately, their use is mostly restricted to research due to their limited access (especially in antigen types used for IFA test). In recent years, DNA amplification after blood culture pre-enrichment became the gold standard for diagnosis of *Bartonella* infection [174]. Treatment of bartonellosis is very difficult, requiring long term treatment with a combination of antibiotics (i.e. azithromycin/minocycline) (e.g. [175–181]. As the pathogens possess a zoonotic potential, prevention of pathogen transmission is essential especially in form of ectoparasite control. This must include also cats as a major reservoir for *Bartonella* spp.

### Lyme borreliosis

Lyme borreliosis (LB) caused by spirochetes of the *Borrelia burgdorferi* (*sensu lato*) species complex is a zoonotic disease affecting humans, dogs, horses and other mammalian species. Vectors in focus are hard ticks of the genus *Ixodes*, but neither the role of the different tick species in the transmission cycle nor the clinical relevance of the different *B. burgdorferi* (*s.l*.) species detected in those tick species in South America is clarified [182–184]. Moreover, a report of the detection of *B. burgdorfei* (*sensu stricto*) in *Dermacentor nitens* ticks in Brazil suggests that the etiology of LB in LATAM is far from being understood [185]. LB has hardly and mainly only based on seroprevalence data been described in pets in LATAM, especially in Mexico [186, 187] and Brazil [38, 188]. Clinical signs in dogs are listed elsewhere [189–194] and only few reports on LB exist in cats [195–198]; for more detailed data see Pantchev et al. [198]. The clinical diagnosis of borreliosis in dogs is very difficult since compatible clinical symptoms with other vector-borne pathogens are very common. Direct detection methods (PCR and/or culture) are difficult and of little practical relevance as the organisms are rarely detected in body fluids [199–201]. Regarding serological diagnosis, detection of specific antibodies does not necessarily correlate with the presence of clinical disease [189]. The method of choice for serological diagnosis is a two-tiered laboratory test [202], consisting of an enzyme-linked immunosorbent assay (ELISA) and immunoblotting (Western blotting); for more detailed information see also Krupka & Straubinger [189]. Furthermore, a commercial ELISA based on C6 peptide is also widely used for serodiagnosis (see Krupka & Straubinger [189] for additional information and further literature). Treatment of LB should be initiated as early as possible [189]. Whether dogs (or cats) should be treated when specific antibodies are detected in the absence of clinical signs is controversial [203–205]. Treatment is recommended for a period of 28 to 30 days, and the most commonly used drug is doxycycline. For further information on treatment regimens etc., see Krupka & Straubinger [189]. Again, prevention of pathogen transmission by ectoparasiticidal control is an essential aspect, especially also because of the zoonotic potential of the pathogens.

### Ehrlichiosis

Ehrlichiosis in dogs and cats has been reported in LATAM. The causative agents are *Ehrlichia canis* (responsible for canine monocytic ehrlichiosis [CME]), *Ehrlichia chaffeensis* and *Ehrlichia ewingii*, with ticks as the transmitting vectors [206–208]. Clinical signs of CME are very similar to the ones presented in granulocytic anaplasmosis and partly also occur in cats. *Ehrlichia ewingii* infection is also reported to go along with clinical signs in dogs, but none in cats, whereas *E. chaffeensis* infection usually presents mildly or subclinically unless present in co-infection, and again with no reported signs in cats. For more details on CME see Sainz et al. [98] and on all three pathogens see Allison & Little [209]. Detection of *E. canis* morulae (an aggregate of *E. canis* organisms) in a blood smear, ideally a buffy coat smear, is indicative, but rather rare in clinical cases [210]. Further diagnostic tests, such as serology or molecular techniques (PCR) must be performed. CME can be diagnosed with IFA test or ELISA [211–213]. A fourfold increase in IgG antibodies over time has been suggested to be taken as evidence of an ongoing infection [213], as well as the combination of serology and PCR has been recommended for diagnosis of infection [214]. Nevertheless, use of some of these test systems might not be available for whole of LATAM. Additionally, rapid serological tests are available; for more detailed information on diagnostics see also Sainz et al. [98] and Allison & Little [209]. Doxycycline is considered the treatment of choice for rickettsial infections [100, 215, 216], thus also for ehrlichiosis; for details on the treatment regimen see among others Allison & Little [209] and Sainz et al. [98]. Again, avoidance of tick exposure and prevention of transmission by use of ectoparasiticidal compounds are essential. This is of vital importance as the mentioned pathogens may have zoonotic character (Venezuela [217], LATAM [218–223]).

### Hemotropic mycoplasmosis

Hemotropic mycoplasmosis (formerly known as hemobartonellosis) has rarely been reported in LATAM. The disease in dogs is caused mainly by *Mycoplasma haemocanis* and *Mycoplasma haematoparvum*. In cats, the disease can be caused by single- or co-infections with *Mycoplasma haemofelis*, *Mycoplasma haemominutum* and *Mycoplasma turicensis*. Blood transfusions have been reported as a source of infections (e.g. [224, 225]), but blood-sucking arthropods are likely to be involved in the transmission as well [226–231]. Generally, little is known on the ecology and form of transmission of these bacteria. Clinical signs may vary and are listed elsewhere [232, 233]. Specific conventional and quantitative real-time PCR systems have been introduced and are now considered the gold standard [234–239]. Treatment is performed depending on the severity of the infection. Antibiotics such as doxycycline or tetracycline should be effective, but consistent clearance of infection was not seen with a range of antibiotics [233]; for more details on treatment see among others Messick [233] and Willi et al. [240]. As with all potentially vector-transmitted pathogens, prevention in form of vector control is essential.

### Rickettsiosis

Rickettsiosis has long been associated only with tick-borne *Rickettsia* species from the spotted fever group, with two very prominent representatives: *Rickettsia rickettsii* [agent of Rocky Mountain spotted fever (RMSF) and Brazilian spotted fever (BSF), also called fiebre manchada in Mexico and febre maculosa in Brazil] [241] and *Rickettsia conorii* [agent of Mediterranean spotted fever (MSF) or Boutonneuse fever] [242]. Meanwhile several further species have been identified as human and partly also companion animal pathogens, which are not only tick-borne (e.g. *Rickettsia massiliae*, *Rickettsia parkeri*, *Rickettsia felis*). Several tick species, among others from the genera *Amblyomma*, *Dermacentor* and *Rhipicephalus*, but also flea species from the genera *Ctenocephalides* and *Archeopsylla*, have been identified as vectors for the above-mentioned different *Rickettsia* species [243]. Infection of dogs and cats with *Rickettsia* species is often subclinical, inapparent, but may also result in severe disease (especially in the case of *R. rickettsii*) [244], potentially being even fatal [245]. For an overview on the different *Rickettsia* species see also Nicholson et al. [215] and Allison & Little [209]. Diagnosis of rickettsial pathogens is usually achieved by PCR assays, serological assays or response to treatment in most clinical cases. When PCR is not practical or available, serology, and here particularly documentation of seroconversion in an acutely ill individual, should be used. For detailed information on the different diagnostic approaches in *Rickettsia* spp. see also Allison & Little [209]. The antibiotic treatment of choice is doxycycline [215, 246]. Prompt treatment is critical as delays can result in fatality [209]. Besides the clinical effect of some *Rickettsia* species in dogs, dogs are important sentinels of infection and disease (e.g. in *R. conorii*) [247, 248]. They are also expected to play an important role as biological hosts of the ticks and serve to increase the infected tick population in close association with human habitation (again for *R. conorii*) [215]. Thus, ectoparasitic control is essential also under the zoonotic aspect and the concept of One Health.

At the end of the presentations of the relevant VBDs we want to remark that veterinarians should be aware of synergistic effects and clinically relevant immunosuppression in co-infected animals [249] as well as an altered clinical appearance in co-infected animals, potentially making diagnosis more difficult and probably leading to a more serious disease outcome [250]. This is relevant for the whole LATAM region as exposure to several pathogens seems possible.

## Country files

Subsequently a listing of occurrence of the pathogens respectively of corresponding seroprevalence data in LATAM by country in alphabetical order follows, based on an actual literature search. Additionally, all described data are summarized in Table 1.

**Table 1:** Tabular overview on the occurrence of CVBDs in dogs, cats, humans and wild carnivores in LATAM based on an actual literature search (partly only based on seroprevalence data; single case reports included; questionable cross-reactivities neglected)

Country	Host	Bab	HWD	SD	Hep	Leish^a^	Tryp^b^	Ana	Bart	Bor	Ehr	Myc	Rick
Argentina	Dogs	Y	Y	–	Y	Y	Y	Y	Y	–	Y	Y	–
	Cats	–	–	–	–	–	Y	–	Y	–	–	–	–
	Humans	–	–	–	–	Y (CL, VL)	Y	–	–	Y	Y	–	Y
	Wild carnivores	–	–	–	Y	–	–	–	–	–	–	Y	Y
Belize	Dogs	–	–	–	–	–	–	–	–	–	–	–	–
	Cats	–	–	–	–	–	–	–	–	–	–	–	–
	Humans	–	–	–	–	Y (CL)	Y	–	–	–	–	–	–
Bolivia	Dogs	–	Y	–	–	Y	Y	–	–	–	Y	–	Y
	Cats	–	–	–	–	–	–	–	–	–	–	–	–
	Humans	–	–	–	–	Y (CL, VL)	Y	–	–	Y	–	–	–
Brazil	Dogs	Y	Y	–	Y	Y	Y	Y	Y	Y	Y	Y	Y
	Cats	Y	–	–	–	Y	Y	Y	Y	–	Y	Y	–
	Humans	–	–	–	–	Y (CL, VL)	Y	–	–	Y	–	Y	Y
	Wild carnivores	–	–	–	–	–	–	–	Y (in captivity)	–	–	Y (in captivity)	
Caribbean Islands	Dogs	Y	Y	–	Y	–	Y	Y	Y	–	Y	Y	–
	Cats	Y	Y	–	–	–	–	–	Y	–	Y	Y	–
	Humans	–	–	–	–	Y (CL, VL)	–	–	–	–	–	–	–
Chile	Dogs	–	–	Y	–	–	Y	Y	Y	–	Y	–	Y
	Cats	–	–	–	–	–	Y	–	Y	–	–	–	–
	Humans	–	–	Y	–	–	Y	–	–	Y	–	–	–
	Wild carnivores	–	–	–	–	–		–	–	–	–	Y	Y
Colombia	Dogs	Y	Y	–	Y	Y	Y	Y	Y	–	Y	–	Y
	Cats	–	–	–	–	–	–	–	–	–	–	–	–
	Humans	–	–	–	–	Y (CL, VL)	Y	–	–	Y	–	–	–
Costa Rica	Dogs	Y	Y	–	Y	–	Y	Y	–	Y	Y	–	Y
	Cats	–	–	–	–	–	–	–	–	–	–	–	–
	Humans	–	–	–	–	Y (CL)	Y	–	–	–	–	–	–
Cuba	Dogs	–	Y	–	–	–	–	–	–	–	–	–	–
	Cats	–	–	–	–	–	–	–	–	–	–	–	–
	Humans	–	–	–	–	–	–	–	–	Y	–	–	–
Dominican Republic	Dogs	–	Y	–	–	–	–	–	–	–	–	–	–
	Cats	–	–	–	–	–	–	–	–	–	–	–	–
	Humans	–	–	–	–	Y (CL)	–	–	–	–	–	–	–
Ecuador	Dogs	Y	Y	–	–	Y	Y	Y	Y	–	Y	Y	–
	Cats	–	Y	–	–	–	–	–	Y	–	–	Y	–
	Humans	–	–	–	–	Y (CL)	Y	–	–	–	–	–	–
El Salvador	Dogs	–	Y	–	–	–	Y	–	–	–	–	–	–
	Cats	–	–	–	–	–	Y	–	–	–	–	–	–
	Humans	–	–	–	–	Y (CL, VL)	Y	–	–	–	–	–	–
French Guiana	Dogs	–	–	–	–	Y	–	Y	–	–	Y	–	–
	Cats	–	–	–	–	Y	–	–	–	–	–	–	–
	Humans	–	–	–	–	Y	Y	–	–	–	–	–	–
Guatemala	Dogs	–	–	–	–	Y	Y	–	–	–	–	–	–
	Cats	–	–	–	–	–	–	–	Y	–	–	–	–
	Humans	–	–	–	–	Y (CL, VL)	Y			–	–	–	–
Guyana (British Guyana)	Dogs		Y	–	–	–	–	–	–	–	–	–	–
	Cats	–	–	–	–	–	–	–	–	–	–	–	–
	Humans	–	–	–	–	Y (CL)	Y	–	–	–	–	–	–
Honduras	Dogs	–	Y	–	–	Y	Y	–	–	–	Y	–	–
	Cats	–	–	–	–	Y	Y	–	–	Y	–	–	Y
	Humans	–	–	–	–	Y (CL, VL)	Y	–	–	–	–	–	–
Mexico	Dogs	–	Y	Y	–	Y	Y	Y	–	Y	Y	–	Y
	Cats	–	–	–	–	Y	Y	–	–	–	–	–	–
	Humans	–	–	–	–	Y (CL, VL)	Y	–	–	Y	–	–	Y
Nicaragua	Dogs	Y	Y	–	Y	–	–	Y	–	–	Y	–	Y
	Cats	–	–	–	–	–	–	–	–	–	–	–	–
	Humans	–	–	–	–	Y (CL, VL)	Y	–	–	–	–	–	–
Panama	Dogs	–	–	–	–	Y	Y	Y	–	–	Y	–	Y
	Cats	–	–	–	–	–	–	–	–	–	–	–	–
	Humans	–	–	–	–	Y (CL)	Y	–	–	–	–	–	–
Paraguay	Dogs	Y	Y	–	–	Y	Y	Y	–	–	Y	–	–
	Cats	–	–	–	–	–	Y	–	–	–	–	–	–
	Humans	–	–	–	–	Y (CL, VL)	Y	–	–	–	–	–	–
Peru	Dogs	–	Y	–	–	Y	Y	Y	Y	Y	Y	–	Y
	Cats	–	–	–	–	–	–	–	Y	–	–	–	Y
	Humans	–	–	–	–	Y (CL)	Y	–	–	Y	Y	–	Y
Puerto Rico	Dogs	–	Y	–	–	–	–	Y	–	–	Y	–	–
	Cats	–		–	–	–	–	–	–	–	–	–	–
	Humans	–	Y	–	–	–	–	–	–	–	–	–	–
Suriname	Dogs	–	Y	–	–	Y	Y	–	–	–	–	–	–
	Cats	–	–	–	–	–	–	–	–	–	–	–	–
	Humans	–	–	–	–	Y (CL)	Y	–	–	–	–	–	–
Uruguay	Dogs	–	–	–	–	Y	–	Y	–	–	–	–	Y
	Cats	–	–	–	–	–	–	–	–	–	–	–	–
	Humans	–	–	–	–	–	Y	–	Y	–	–	–	Y
Venezuela	Dogs	Y	Y	–	Y	Y	Y	Y	–	–	Y	–	–
	Cats	–	Y	–	–	–	–	–	–	–	–	–	–
	Humans	–	–	–	–	Y (CL, VL)	Y	–	–	Y	–	–	–

^a^In case of human visceral and cutaneous leishmaniosis, if not cited otherwise within the additional files, status for humans is according to the WHO webpages [708, 709]

^b^In case of human trypanosomosis, if not cited otherwise within the additional files, status for humans is according to the WHO webpage [710]

*Abbreviations*: Y, yes (reported); –, not reported; Bab, babesiosis; HWD, heartworm disease; SD, subcutaneous dirofilariosis; Hep, hepatozoonosis; Leish, leishmaniosis; Tryp, trypanosomosis; Ana, anaplasmosis; Bart, bartonellosis; Bor, borreliosis; Ehr, ehrlichiosis; Myc, mycoplasmosis; Rick, rickettsiosis; CL, cutaneous leishmaniosis; VL, visceral leishmaniosis

### Argentina

#### Parasitic diseases

As in many countries in LATAM, the most common parasitic diseases reported in Argentina are trypanosomosis (responsible for Chagas disease in humans), dirofilariosis and leishmaniosis.

Babesiosis due to *B. vogeli* has been described in three dogs from Buenos Aires [12, 251] and detected in 10% (2/21) and 6.8% (3/41) of shelter dogs from Córdoba and Santa Fé, respectively, by molecular methods [252]. Large piroplasms have furthermore been detected in 0.2% of tested animals in a large canine survey with more than 16,000 dogs [12, 251]. *Babesia vogeli* was also detected in cat fleas (*Ctenocephalides felis*) collected from shelter dogs in Córdoba and Santa Fé (R. Maggi, unpublished data). Interestingly, *Babesia* was not detected in any of 48 free ranging Pampas gray foxes (*Lycalopex gymnocercus*) from Rio Negro that showed high prevalence for hepatozoonosis [253].

Dirofilariosis caused by *D. immitis* has been reported in Buenos Aires [254–256] and Mendoza [257]. Epidemiological studies in Argentina suggest that the prevalence of dirofilariosis in dogs is highly variable, showing a significantly heterogeneous temporal and spatial distribution [254–256, 258, 259]. In Buenos Aires, screening of 19,298 blood samples from 65 localities showed prevalence values of 1.63% by microhematocrit tube technique, 3.65% by modified Knottʼs test, and 14.41% by antigen test [255].

Hepatozoonosis has been reported in dogs (infected with *H. canis*) from Buenos Aires [251, 260], and in up to 50% of 48 blood samples from free ranging Pampas gray foxes (*L. gymnocercus*) from Rio Negro (infected with *Hepatozoon* sp.) [253, 261]. *Hepatozoon* sp. infection has further been described in single canine cases in the Buenos Aires region [262]. No prevalence studies are available up to date.

For leishmaniosis, only few records are available regarding the overall prevalence in Argentina. *Leishmania braziliensis* and *L. infantum* have been associated with canine leishmaniosis in several provinces of the country, including Entre Rios, Santa Fé, Misiones, Chaco, Salta and Santiago del Estero [263–270]. Reports from Misiones, which represents one of the areas with highest endemicity for the disease in Argentina, indicate prevalences as high as 57% in dogs (43.6% seropositive and 47.3% positive by PCR) [266]. In other provinces, i.e. Salta, a significant seroprevalence (13.0–27.4%) has also been reported [263, 268].

Trypanosomiasis is one of the most important endemic VBDs in Argentina. Serological surveys in the northern rural regions have shown prevalences in dogs ranging between 23–84%; while seroprevalence in cats has been reported at 28.7% [83, 263, 271–277]. In hyperendemic regions, such as Chaco, molecular prevalence as high as 53% has been reported in dogs [278].

#### Bacterial diseases

Anaplasmosis due to *A. platys* infection was reported in prevalences ranging between 13.5–37.5% in sick dogs from Buenos Aires [251, 279, 280] detected by molecular techniques, and in 12.5% and 17.4% of dogs from Cordóba and Santa Fé [252], respectively. No data are available from other provinces. Nevertheless, *A. platys* was detected in *R. sanguineus* (*s.l.*) ticks from Chaco Province [281], and from cat fleas (*C. felis*) collected from shelter dogs in Córdoba and Santa Fé (R. Maggi, unpublished data).

Bartonellosis due to *B. vinsonii berkhoffii* has been detected in dogs with endocarditis in Buenos Aires (R. Maggi, unpublished data). *Bartonella* infection has been detected at a molecular prevalence of 3% in shelter dogs from Córdoba (close homology to *B. tribocorum*), and from Santa Fé (*B. clarridgeiae*). *Bartonella clarridgeiae* has also been detected in cat fleas (*C. felis*) collected from shelter dogs in Córdoba and Santa Fé (R. Maggi, unpublished data). Additionally, *B. henselae* and *B. clarridgeiae* have been detected at a molecular prevalence of 17.8% in cats from Buenos Aires [282].

Lyme borreliosis in dogs or cats in Argentina has not been reported yet. Nevertheless, the detection of *B. burgdorferi* (*s.l*.) infecting ticks in northern provinces [184], as well as the detection of antibodies against *B. burgdorferi* in farm workers has been reported [283].

Ehrlichiosis due to *E. canis* has been reported at a molecular prevalence in 7% of sick dogs from Buenos Aires [251]. No data are available on detection or prevalence of *Ehrlichia* spp. infecting dogs from other provinces, although *E. canis* was detected in *R. sanguineus* (*s.l*.) ticks from Formosa Province [281]. *Ehrlichia chaffeensis* has been found at a prevalence of 14% in people from Jujuy [221] and detected in *A. parvum* ticks collected from several mammal species (including a dog and humans) from Santiago del Estero [208].

Hemotropic mycoplasmosis mainly due to infection with *M. haemocanis* or *M. haematoparvum* has been detected at molecular prevalences of 83.3% and 73.9% in shelter dogs from Córdoba and Santa Fé, respectively [252]. Similarly, both pathogens were also detected in cat fleas (*C. felis*) collected from shelter dogs in Córdoba and Santa Fé (R. Maggi, unpublished data). Other species (*Mycoplasma suis*) have also been described in dogs [252]. Hemotropic mycoplasmas were also detected in up to 8.3% of 48 blood samples from free ranging Pampas gray foxes (*L. gymnocercus*) from Rio Negro [253].

Rickettsiosis has not been reported in dogs or cats yet in Argentina, but in 2.1% from 48 blood samples from free ranging Pampas gray foxes (*L. gymnocercus*) from Rio Negro [253]. Cases of human rickettsiosis due to *R. rickettsii* and *R. parkeri* infection have been reported in Jujuy and Buenos Aires [221, 284–287]. *Rickettsia* species have been reported in several tick species: *R. parkeri* and *R. bellii* in *Amblyomma triste* from Entre Rios, Santa Fé, Córdoba, Buenos Aires, La Rioja, and in other northern provinces, and *R. massiliae* in *R. sanguineus* (*s.l*.) in Buenos Aires [279, 280]. Meanwhile *R. felis* has been detected in single cat fleas (*C. felis*) collected from dogs [288].

### Belize

Data on VBDs in pet animals from Belize are very scarce or not existent.

#### Parasitic diseases

Leishmaniosis and trypanosomosis are the only two VBDs reported in people and vectors and as such their pathogens could be recognized as potential infectious agents for pets. *Leishmania donovani*, *L. braziliensis* and *L. mexicana* have been reported in people and sand flies [289–295], and meanwhile *Trypanosoma* infection has been reported in people and *Triatoma* species [296, 297].

#### Bacterial diseases

Rickettsiois: spotted fever group rickettsiae, especially *R. amblyommatis* and *R. parkeri*, were detected in *Amblyomma* species among others from dogs, suggesting a risk of tick-borne rickettsioses to humans and animals in Belize [298].

### Bolivia

Data on VBDs in pet animals from Bolivia are very scarce or not existent.

#### Parasitic diseases

Dirofilariosis due to *D. immitis* has been reported in dogs at an average of 33% seroprevalence (range: 22–41%) in different villages [299] and at *c.*10% in the Isoso of Bolivia [300]. Leishmaniosis has been reported in healthy dogs at a seroprevalence of 11.8% [301]. Trypanosomosis due to *T. cruzi* in dogs was detected at a seroprevalence of 9.6% in Santa Cruz [302].

#### Bacterial diseases

Lyme borreliosis has been detected in people in the Santa Cruz department, south-eastern Bolivia, whereas dog sera failed to show positive seroprevalence for this pathogen [303, 304]. Ehrlichiosis due to *E. canis* was reported at a seroprevalence of 86% in domestic dogs [299]. Rickettsiosis in dogs due to *R. rickettsii* was reported at seroprevalences ranging between 68.2–86.0% [299, 305], while antibodies against *R. parkeri* were detected in 2.3% of dogs from Cochabamba [305]. Rickettsial species were also detected in *Amblyomma* ticks (*Amblyomma tigrinum*) [305].

### Brazil

A comprehensive review on VBDs has been published by Dantas-Torres [38].

#### Parasitic diseases

Babesiosis due to *B. vogeli* has been recognized in Brazil since the beginning of the 20th century. *Babesia gibsoni* infection in dogs has also been reported virtually in all Brazilian regions. The reported seroprevalence of infection in dogs ranges between 35.7–72.0% [38, 306–314]. In cats, *B. vogeli* has been reported at a molecular prevalence ranging between 11.9–16.0% [315, 316].

Dirofilariosis: Canine heartworm infections due to *D. immitis* are frequently reported in Brazil with prevalences that range from 2% to up to 23.1% [38, 174, 317–320].

Hepatozoonosis due to *H. canis* is present in almost all regions. Prevalences of 39.2–58.8% have been reported in rural and urban areas [38, 39, 307, 321, 322].

Leishmaniosis was firstly recognized in Brazil during the 1930s. Canine visceral leishmaniosis by *L. infantum* is endemic in all Brazilian regions, meanwhile also occurring in the South of the country [38, 306, 323–326]. Canine cutaneous leishmaniosis is also prevalent in all regions with prevalences ranging between 3.2–50.3%, depending on the area and methods of diagnosis used [323, 327–335]. The seroprevalence of *Leishmania* infection in dogs varies widely and can be as high as 67% in highly endemic foci [336]. In cats, seroprevalence of 54% has been also reported [337].

Trypanosomosis has been reported in almost all areas of Brazil. In areas where American trypanosomosis (or Chagas disease) is endemic, seroprevalences to *T. cruzi* between 16.0–71.6% in dogs were reported [338–340]. Clinically, the infection is of minor significance, as infected dogs are often asymptomatic carriers [38]. In cats, *T. cruzi* seroprevalence of 51% has been reported [337]. *Trypanosoma evansi* infection in dogs is found predominately in the Center-West and the South regions [341–350]. The seroprevalence of infection in dogs with *T. evansi* ranges between 15.7–30.0% [38, 341, 351].

#### Bacterial diseases

Anaplasmosis caused by *A. platys* in dogs is found in all regions according to Dantas-Torres [38] but has only sporadically been published. Molecular prevalences in dogs are ranging between 1.6–48.8% [306, 308, 309, 352, 353]. *Anaplasma phagocytophilum* has been reported at molecular prevalences between 6–7% in dogs [354, 355], 8% in cats [315] and in ixodid ticks [354].

Bartonellosis has been described in dogs and cats in southern Brazil. In sick dogs from southern states, prevalences in dogs of 1.9–3.9% have been reported to infection with *B. vinsonii berkhoffii* and *B. henselae* [324, 356–358]. In addition, *B. vinsonii berkhoffii* and *B. clarridgeiae* were detected by serology in captive wild canids (at seroprevalences ranging between 8–13%) from 19 zoos in São Paulo and Mato Grosso states [359]. In feral cats, the molecular prevalence for *Bartonella* infection can be as high as 17% [360, 361].

Lyme borreliosis has been recognized in humans in Brazil since 1989 [188, 362]. Serological surveys in dogs from Southeast Brazil showed ranges from less than 1% up to 20% [38]; while seroprevalences of up to 51% have been reported from Espirito Santo [188]. The pathogen has been recovered from *Ixodes* spp. (*B. burgdorferi* (*s.l*.) group) and from *D. nitens* ticks (*B. burgdorferi* B31 strain) [185, 363], but the role of the vector and the clinical relevance of the species have yet to be determined.

Ehrlichiosis, due to infection with *E. canis*, was firstly recognized in Brazil in the 1970s, and is prevalent in virtually all regions (for a comprehensive review on ehrlichiosis in Brazil, see Vieira et al. [364]). The seroprevalence of infection varies between the southern, Central-West and northern-northeastern regions of Brazil, but it can be as high as 62.8% in asymptomatic and 78% in symptomatic dogs [38, 306, 309–312, 324, 352, 364–375]. Molecular prevalence for *E. canis* has been found in dogs at a range of 15–88% [316, 364]. Infections in dogs with other *Ehrlichia* species, i.e. *E. chaffeensis* and *E. ewingii*, have also been reported [376]. In cats, *E. canis* or a closely related species have also been reported at a molecular level, with a prevalence ranging between 9.4–20.0% [377, 378].

Hemotropic mycoplasmosis has been recognized in Brazil and has been reported in several wild canids and felids as well as in humans [379–382]. Several species of hemotropic mycoplasmas have been detected in dogs and cats [308, 361, 382–388]. The most predominant species in dogs is *M. haemocanis*, which has been recognized in South and Southeast Brazil. Other species such as *M. haematoparvum*, *M. haemofelis*, *M. turicensis* and *M. haemominutum*, have been detected in neotropical and exotic wild canids and felids from Brazilian zoos, and in feral cats [380]. Molecular prevalence of up to 32% has been reported in cats [315] and prevalences of 7–45% have been reported in dogs [382, 388].

Rickettsiosis due to several species of the spotted fever *Rickettsia* group, has been reported among others in humans and dogs [389–393]. Seroprevalence for *R. rickettsii* in dogs ranges between 2.7–64.0%, while seroprevalence of 2.7–7.3% has been reported for *R. parkeri* [371, 373, 389, 390, 392, 393]. Rickettsial species have also been reported in several tick species of the genera *Amblyomma*, in *R. sanguineus* (*s.l*.), and in cat fleas (e.g. [393–404]).

### Caribbean Islands (excluding Cuba, Dominican Republic and Puerto Rico, listed separately)

The information on vector-borne pathogens on the Caribbean Islands in extremely scarce and fragmented.

#### Parasitic diseases

Babesiosis has been described on several islands. Molecular screening of dogs in St. Kitts showed an overall prevalence of 24% for *Babesia* spp., of which 48% and 40% were due to *B. vogeli* and *B. gibsoni*, respectively, 2% were due to co-infections with both species and in 10% *Babesia* species was unidentified [13]. *Babesia vogeli* was also detected by PCR in 7% of dogs surveyed in Grenada [405] and in dogs in Trinidad [406]. Interestingly, *B. vogeli* infection was also detected in cats in Trinidad by PCR at 6.7% prevalence [406]. Finally, there is anecdotal record on *B. canis* (sp.) infection in dogs in Aruba [407]; *Babesia* infection has been reported by microscopy or serology in dogs visiting the Dutch Antilles [408].

Dirofilariosis has been reported in Turk and Caicos, Curaçao and Grenada. In Grenada, infection with *D. immitis* was documented by microfilarial identification with prevalences ranging between 9.1–26.8% in dogs affected with caval syndrome and submitted for necropsy [409]. Combining the results of four studies on live dogs and five studies on necropsied dogs (*n* = 1,245) between 2002 and 2009, an estimated overall *D. immitis* infection rate of 13.9% is reported [410]. A survey on feral cats, also in Grenada, showed a seroprevalence of 8% for *D. immitis* [411]. In Turk and Caicos, seroprevalence for *D. immitis* was 58% and 8% for feral and pet dogs, respectively [412]. In Curaçao, two canine surveys detected prevalences of 7.2% and 12.8% for female and for male dogs, respectively an overall prevalence of 9.0% (3.4% in feral and 13.5% in pet dogs) [413, 414].

Hepatozoonosis due to *H. canis* was described in St. Kitts at an overall molecular prevalence of 6% [13]. Meanwhile in Grenada, a molecular prevalence of 7% has been reported for dogs [405]. There are also anecdotal data on *H. canis* infection in dogs in Aruba [407].

Leishmaniosis in the Caribbean Islands has been rarely reported in dogs. In Grenada, screening of dogs using antibodies to visceral leishmaniosis failed to detect positives [415]. Nevertheless, leishmaniosis has been described in humans in Martinique [416, 417] and Guadalupe [418].

Trypanosomosis in wild animals and triatomine vectors has been reported since 1960 in Aruba, Curaçao, Jamaica and Trinidad [419, 420]. In Grenada, a seroprevalence of 13.2% and 4.3–6.4% in stray and pet dogs, respectively has been reported [63, 415].

#### Bacterial diseases

Anaplasmosis was detected in the region at a relatively high prevalence. In St. Kitts, a prevalence of 4% in healthy dogs was reported [13, 421]. In Grenada, prevalences of 19.2% (molecular prevalence) and 24% (seroprevalence) were reported for *Anaplasma* species [405, 422]. *Anaplasma* infections have also been reported in dogs in Trinidad [406].

Bartonellosis in cats and dogs has been reported on a few Caribbean Islands. Infections with *B. henselae*, *B. clarridgeiae*, or both have been reported in 51% of pet cats, and in a range of 52–63% in feral cats from St. Kitts [423]. Similarly, 24% of pet cats and 59% of feral cats were positive for one or both species (*B. henselae* and *B. clarridgeiae*) in Trinidad [424]. In dogs, *Bartonella* species have been also detected at a molecular prevalence of 1.4 % for *B. vinsonii berkhoffii*, and at a seroprevalence of 8.2% for *Bartonella* spp. in Grenada [405].

*Ehrlichia* infection in the region has also been reported on several islands. In St. Kitts, an overall (serological and/or PCR) prevalence of 24% has been reported in dogs [13]. In Trinidad, 14.1% (molecular prevalence) and 44.6% (seroprevalence) have been reported for *E. canis* in healthy and stray dogs, respectively [406, 425]. Prevalences ranging from 24.7% (molecular prevalence) to 31% (seroprevalence) have been reported for *Ehrlichia* species in dogs from Grenada [405, 422]. In Turk and Caicos, seroprevalences of 71% and 18% were reported for feral and pet dogs, respectively [412]. In Aruba 4 of 7 dogs were reported to be infected with *E. canis* confirmed by microscopy [407]. *Ehrlichia* infection has further been reported by microscopy or serology in dogs visiting the Dutch Antilles [408]. *Ehrlichia* *canis* has been detected in cats in Trinidad at a molecular prevalence of 6.7% [406].

Hemotropic mycoplasmosis due to *M. haematoparvum* and *M. haemocanis* has been reported in dogs in Trinidad at a prevalence of 8.1% [239]. *Mycoplasma haemofelis* and *M. haemominutum* have been reported in 31.6% and 33.3% of cats in Trinidad [406, 426].

### Chile

#### Parasitic diseases

Dirofilariosis has been described in dogs from a semi-rural district near Santiago. Microscopic and molecular analysis showed that microfilariae, similar to *D. repens*, were present in about 22% of the dogs with (32%) or without (12%) dermatological symptoms or signs compatible with filarial infections [36]. A single human case with a subcutaneous infection of an unidentified *Dirofilaria* sp. is also reported [427].

Hepatozoonosis: There are no reports on dogs or cats, but *Hepatozoon* spp. has been detected in hard and soft ticks from different regions of Chile [428].

Trypanosomosis in people (Chagas disease) has been recognized to exist in seven of the 13 administrative regions of the country [429–443]. The seroprevalence in dogs has been reported to be over 4.6% in the northern areas [430]. In a large periurban survey, 7.9% of cats and 7.0% of dogs were positive by indirect hemagglutination test [439].

#### Bacterial diseases

Anaplasmosis due to *A. platys* has been reported in sick dogs from Santiago at a molecular prevalence of 20% [444]. Other studies revealed a much higher seroprevalence (69%) against *A. phagocytophilum* in dogs exposed to ticks in the same region [445]. It is not clear whether these results are a consequence of serological cross-reaction with *A. platys*. *Anaplasma* species has been aslo detected in soft ticks in Chile [428].

Bartonellosis has been described in cats but not in dogs from Chile even though *Bartonella* (*B. rochalimae*) has been reported in fleas from dogs [123]. In cats, seroprevalence of *B. henselae* is very high (71–73%) in pet cats [446, 447], and even higher (90%) in stray cats [447]. In addition, *B. henselae* and *B. clarridgeiae* were also reported in fleas from cats [447].

Lyme borreliosis has not been described in dogs, even though there is some debate on Lyme disease in Chile [448, 449]. *Borrelia burgdorferi* (*s.l*.) species have recently been detected in *Ixodes stilesi* ticks [183]. The role of this species in the transmission of Lyme borreliosis has yet to be determined. Different *Borrelia* species, some of them closely related to *Borrelia turicatae* and *Borrelia garinii*, have been detected in hard and soft ticks from different regions of Chile [428].

Ehrlichiosis due to *E. canis* has been reported in single canine cases, confirmed by serology and molecular methods [450] or only by serology [451]. Nevertheless, no data are available for the prevalence and distribution of the disease. Seropositivity in single dogs to *E. chaffeensis* has also been reported [223]. In addition, *Ehrlichia* spp. has been detected in soft ticks from the Chañaral region of Chile [428].

Hemotropic mycoplasmosis due to *M. haemocanis*, *M. haemofelis* and a species closely related to *M. turicensis*, has been reported in wild carnivores (Darwin’s foxes) with a prevalence of up to 57% on Chiloé Island [452].

Rickettsiosis due to *R. conorii* has been reported in dogs from Santiago de Chile with a seroprevalence of 35%, but rickettsial species should be confirmed by molecular studies [445]. *Rickettsia felis* has also been reported in wild foxes (Darwin’s foxes) from Chiloé, with a prevalence of 3% [452], in *R. sanguineus* (*s.l*.) ticks from dogs [453], as well as in *C. felis* fleas from dogs and cats and *Ctenocephalides canis* fleas from dogs [454].

### Colombia

#### Parasitic diseases

Babesiosis due to *B. vogeli* has been frequently described in Colombia. Seroprevalence in dogs has been reported at 4.8% in Bogota, 58% in Villavicencio and 71.8% in Bucaramanga [455].

Dirofilariosis due to *D. immitis* has been reported at prevalences of 1.6% (seroprevalence) [456], 4.8% (Knottʼs test) [457], and 3.8% (Knottʼs test) to 4.6% (Knottʼs test plus antigen ELISA) [458]. In the Colombian Amazon in two Tikuna Indian communities 53.8% (7/13 dogs) of the tested dogs were positive for *D. immitis* by modified Knottʼs test [459].

Hepatozoonosis by *H. canis* has been reported in 31.8% of dogs in the central-western region by molecular and/or microscopic methods [460].

Leishmaniosis in dogs has been frequently described in Colombia. Overall prevalence of infection averaging 33.6% has been reported in northern territories [461], while a seroprevalence of 44.1% (by IFA test) to 50.2% (by ELISA) has been reported from Tolima [462]. Interestingly, a very low seroprevalence (1.6%) has been reported in dogs from Bogota using IFA test [325]. Pathogens of cutaneous leishmaniosis (*L. panamensis*, *L. braziliensis*) have been reported in humans in several areas of Colombia [463–471].

Trypanosomosis has been known to be present in Colombia. In dogs, seroprevalence of 71.6% on Margarita Island [338] and molecular prevalence of 31% for *T. cruzi* in dogs from the Northeast has been reported [472].

#### Bacterial diseases

Anaplasmosis due to *A. platys* has been detected by serology in 53% of dogs from Barranquilla [473]. Additionally, two single *A. phagocytophilum* seropositive dogs have been detected in the same study [473]. Further reports of anaplasmosis due to *A. phagocytophilum* have been published for Colombia at an average seroprevalence of 33% (12% for Medellin, 40% for Barranquilla and 51% for Cartagena) using rapid tests [456]. Nevertheless, caution should be considered regarding cross-reactivity with *A. platys* in this data.

Bartonellosis has been detected at a seroprevalence of 10% in dogs from Bogota testing against *B. vinsonii berkhoffii*, *B. clarridgeiae* and *B. henselae* antigens [356].

Lyme borreliosis has not been detected in dogs [456], even though the disease has been detected in people from rural areas of Colombia [474].

Ehrlichiosis due to *E. canis* has been reported in Colombia at an average seroprevalence of 22% (26% in Medellin, 67% in Bogota, 74–83% in Barranquilla, 80% in Cartagena, 83.9% in Villavicencio and 89.7% in Bucaramanga) [455, 456, 473]. Molecular prevalence for *E. canis* has been reported in Villavicencio at 45.2%, and in Bucaramanga at 59% [455]. Interestingly, a serological survey in rural areas near Bogota showed a 31.8% seroprevalence against *E. chaffeensis* in dogs [475]. Nevertheless, caution should be considered for cross-reactivity with *E. canis*.

Rickettsiosis due to *R. rickettsii* has been reported at a seroprevalence of 18.2% in dogs from rural areas near Bogota [475]. Seropositivity to spotted fever group rickettsiae was also detected in 40.7% of tested dogs in the Caribbean region of Colombia [476]. *Rickettsia amblyommii* respectively “*Candidatus* Rickettsia amblyommii” has been detected by PCR in *Amblyomma cajennense* ticks close to the Colombian border in Panama and in Colombia (Villeta) itself [477, 478], while *R. bellii* and *R. felis* have been detected in *Amblyomma ovale* ticks and in fleas (*C. felis*, *C. canis* and *Pulex irritans*) collected from domestic animals and small mammals [476], respectively, from dogs and cats [479].

### Costa Rica

#### Parasitic diseases

Babesiosis in dogs due to *B. vogeli* was reported at an overall molecular prevalence ranging between 2.4–20.0% [40, 41]. Interestingly, the prevalence varied significantly depending on regions [40]. Babesiosis due to *B. gibsoni* has also been reported in Costa Rica at a molecular prevalence of 5% [41].

Dirofilariosis in dogs due to *D. immitis* infection has been reported at prevalences of 2.3–11.0% (by serology) and 22.6% (by molecular methods) [41, 480–482]. Positive rates were strongly dependent on region, climate, and test system used. The influence of the test system used was especially demonstrated in studies by Rojas et al. [481].

*Hepatozoon* infection in dogs due to *H. canis* was reported at an overall molecular prevalence ranging between 2.4–37.5% [40, 41] with huge differences between the tested regions [40].

Leishmaniosis has not been detected in surveys of dogs from the regions central, Pacific and Atlantic [40].

Trypanosomosis due to *T. cruzi* has been reported in dogs from Costa Rica at a seroprevalence ranging between 1.6–27.7% [85, 483–485].

#### Bacterial diseases

Anaplasmosis due to *A. platys* has been reported in Costa Rica at a molecular prevalence ranging between 1–10% in dogs [40, 41, 486–488], with obvious differences between the tested regions [40]. *Anaplasma phagocytophilum* has been reported in single canine cases by PCR [486, 489] and at a seroprevalence of 2.7% [486] and 3.8% [490], with questionable capability to differentiate between the two pathogen species in the latter study. Finally, Montenegro et al. [482] reported an overall seroprevalence in all seven provinces for *Anaplasma* spp. of 6.4%, with no differentiation between the two species due to cross-reactions in the test system used.

Bartonellosis was not reported in dogs or cats in Costa Rica. Nevertheless, *B. clarridgeiae* and *B. henselae* have been detected in cat fleas, whereas *B. vinsonii berkhoffii* and *B. rochalimae* have been detected in dog fleas [491].

Lyme borreliosis in form of seropositivity to *B. burgdorferi* (*s.l*.) antigen has been documented in a single dog from Costa Rica [482] with questionable autochthonous character. A further single seropositive canine case without a proof of an actual infection by PCR has been reported [492].

Ehrlichiosis due to *E. canis* has been reported from Costa Rica at a molecular prevalence ranging between 3.2–50.0% [40, 41, 493, 494]. Interestingly, *E. canis* prevalence varies massively depending on the region [40]. Seroprevalence in dogs for *E. canis* has been reported at a range of 3.5–38.2% [480, 482, 490, 494]. Furthermore, *E. chaffeensis* has been detected at a molecular prevalence of 59% in dogs [495].

Rickettsiosis due to *R. rickettsii*, *R. amblyommii*, *R. felis*, *R. rhipicephali* and *R. parkeri* has been reported at varying seroprevalences in dogs from San Jose [496]. Furthermore, *R. felis* has been detected in cat fleas [497, 498] and *R. amblyommii* has been detected in *A. cajennense* ticks [497].

### Cuba

The information on vector-borne pathogens on Cuba is very scarce and fragmented.

#### Parasitic diseases

Dirofilariosis due to *D. immitis* was reported on Cuba in a range between 6.7–40.0% in dogs [499–501].

#### Bacterial diseases

Lyme borreliosis: The disease has not been officially reported in Cuba. However, clinical cases resembling Lyme disease and serologically positive cases have been reported in humans [502, 503], but existence of *B. burgdorferi* (*s.l*.) is still much debated [504, 505]. No prevalence data for dogs or cats are available for the region.

### Dominican Republic

The information on vector-borne pathogens in the Dominican Republic is extremely scarce or non-existent.

#### Parasitic diseases

Dirofilariosis by antigen detection or microfilaria evidence in dogs has been reported at a prevalence of 18.2% on Samana Peninsula [506] and at a prevalence of 18% in Santo Domingo [507]. An autochthonous focus for cutaneous leishmaniosis in humans has been described within the last 20 years is the Dominican Republic [508–511]. Nevertheless, no prevalence data for dogs are available.

### Ecuador

#### Parasitic diseases

Babesiosis due to *Babesia* spp. has been reported in dogs from Cuenca (by blood smear analysis) at a prevalence of 40.6% [512]. No *Babesia* spp. antibodies were detected in a screening for different *Babesia* species of dogs on Isabela Island, Galapagos [7].

Dirofilariosis has been reported only on Isabela Island, Galapagos, with 34% seroprevalence in dogs and 2% in cats [7].

Leishmaniosis in dogs was reported on the Pacific coast of Ecuador and in other areas [513, 514]. Seroprevalence of 4% against *L. donovani* was also detected in dogs on Isabela Island, Galapagos [7].

Trypanosomosis in people (Chagas disease) was described in Ecuador in 1930 in the province of Guayas and thereafter in various other provinces [515–522]. A serosurvey on dogs, performed in two towns in Guayas province detected seroprevalences of 9.1% and 14.3%, determined by ELISA [518]. *Trypanosoma* infection was not detected in dogs or cats from Isabela Island, Galapagos [7].

#### Bacterial diseases

Anaplasmosis due to *A. platys* was reported in a single dog from Isabela Island, Galapagos [7]. *Anaplasma phagocytophilum* was reported by blood smear analysis in Cuenca at a prevalence of 3.1% [512] and by rapid test at different seroprevalences (26–48%) in dogs in Manta and Guyaquil [523], but cross-reaction with *A. platys* especially in the latter survey should be borne in mind. In addition, an *Anaplasma* species closely related to *A. phagocytophilum* was described in *Amblyomma multipunctum* and *Rhipicephalus microplus* ticks collected from the Antisana Ecological Reserve and Cayambe-Coca National Park [524].

Bartonellosis was detected on Isabela Island, Galapagos, at a seroprevalence of 75% in cats, and at a molecular prevalence of 13% in dogs [7]. The most common species identified by DNA amplification in cats were *B. henselae* and *B. clarridgeiae*, while *B. henselae, B. clarridgeiae*, and *B. elizabethae* were detected in dogs [7].

Lyme borreliosis was not detected in dogs surveyed on Isabela Island, Galapagos [7].

Ehrlichiosis due to *E. canis* estimated by blood smear analysis has also been reported from Cuenca at a prevalence of 56.3% [512] and by rapid test at different seroprevalences (66–78%) in dogs in Guyaquil and Manta [523]. *Ehrlichia* infection (determined by IFA test or PCR) was not detected in dogs from Isabela Island, Galapagos [7].

Hemotropic mycoplasmosis has been reported at a molecular prevalence of 2% in cats and of 1% in dogs on Isabela Island, Galapagos [7].

Rickettsiosis has not been reported in dogs or cats, although a *R. felis*-like organism was identified in *C. felis* fleas collected from dogs from Pastaza and Chimborazo provinces [525].

### El Salvador

The information on vector-borne pathogens in El Salvador is extremely scarce or non-existent.

#### Parasitic diseases

Dirofilariosis: Infection with *D. immitis* has been described in dogs from northern El Salvador at a seroprevalence of 3% [526]. In a study performed on dogs from the coastal areas of El Puerto de La Libertad (La Libertad), prevalences ranging between 11–19%, depending on the type of methods used, were detected [527].

Leishmaniosis: The pathogen of visceral leishmaniosis *L. infantum* (syn. *L. chagasi*) has been isolated in a human case of cutaneous leishmaniosis in El Salvador [528], but no data on dogs or cats are available.

Trypanosomosis has been known to be present in El Salvador affecting people as Chagas disease since 1913. In 1976, prevalences (by xenodiagnosis) of 5% and 7.1% were reported for *T. cruzi* and *T. rangeli* in dogs, respectively [529], while prevalence values of 1.4% and 4.2% were reported for the same species, respectively, in cats [529].

### French Guiana

As mentioned beforehand to a number of countries, the information on CVBDs in French Guiana is very scarce.

#### Parasitic diseases

Leishmaniosis has been widely reported in people [530–537]. Only two canine cases (one with questionable autochthonous character) and one clinical case of cutaneous leishmaniosis due to *L. braziliensis* in a domestic cat have been reported [538, 539]. Trypanosomosis has been known to be present in French Guiana affecting people (Chagas disease) [540–546].

#### Bacterial diseases

Anaplasmosis has been described in dogs from French Guiana at a molecular prevalence for *A. platys* of 15.4% [547]. Ehrlichiosis due to *E. canis* has been reported at a seroprevalence of 6.6% in dogs imported from French Guiana to France [548]. No other data are available for *Ehrlichia* species prevalence in the region.

### Guatemala

The state of knowledge is very scarce for CVBDs in Guatemala.

#### Parasitic diseases

Leishmaniosis has been reported in the Peten Region with a seroprevalence of 28% in dogs [549]. Trypanosomosis has been described in dogs at a seroprevalence of 37% [550].

#### Bacterial diseases

Bartonellosis due to *Bartonella* species has been reported in cats [551], but not in dogs. Rickettsiosis has not been reported in Guatemala in dogs or cats. Nevertheless, *R. felis* has been reported in cat fleas [498].

### Guyana (British Guyana)

The information on vector-borne pathogens in Guyana is extremely scarce or non-existent.

#### Parasitic diseases

Dirofilariosis by *D. immitis* was reported in 1964 at an overall prevalence of 14.1% in 2135 dogs screened *via* Knottʼs test [552]. Leishmaniosis in people has been described frequently [553–555], but no published data on dogs or cats are available. Trypanosomosis due to *T. cruzi* has been sporadically reported in humans [542]. No reports on infection or prevalence are available for dogs or cats.

### Honduras

#### Parasitic diseases

Dirofilariosis has been detected and reported in dogs from Roatán, Islas de la Bahía, at a prevalence of 30% (Knottʼs test) [556].

Leishmaniosis due to *L. donovani* was detected at a seroprevalence of 25% in cats [557] and ranging between 1.4–8.6% in dogs [557–560], but caution should be taken due to cross-reactivity with *T. cruzi*. Visceral and cutaneous leishmaniosis have been reported in humans in Honduras for some time with *L. chagasi* and *L. mexicana* as underlying pathogens [560, 561].

Trypanosomosis in people due to *T. cruzi* and *T. rangeli* is present in Honduras [558, 559]. A study in cats revealed a 16% prevalence rate for *T. cruzi* [557]. Although official reports on trypanosomosis in dogs are not available, apart from a single described canine isolate by Acosta et al. [558], the presence of the disease in dogs has been suggested due to the serological cross-reactivity between *T. cruzi* and *L. donovani* [559].

#### Bacterial diseases

Lyme borreliosis in form of seropositivity has been detected in cats at 25% prevalence [557]. Ehrlichiosis has been detected in dogs at a molecular prevalence of 23.7% for *E. canis*. Ticks collected from dogs have also been tested positive for *E. canis* [562]. Rickettsiosis due to *R. rickettsii* has been reported in cats at 16% seroprevalence [557].

### Mexico

#### Parasitic diseases

Babesiosis has been described in 3 of 22 sick dogs from Morelos (13.6%), using DNA amplification [563] and in 3 of 30 dogs from Veracruz (10%) using indirect haemagglutination test [564].

Dirofilariosis has been described in dogs from all regions. Prevalence of 1.3% in central Mexico, 60% in Celestum, and 8.3% in Yucatan for *D. immitis* have been reported [565–567]. *Dirofilaria repens* has also been reported in a single dog in Guanajuato [35].

Leishmaniosis was described to affect dogs and cats in several regions. In dogs, seroprevalences ranging between 7.5–32.8% for *L. braziliensis*, 4.7–41.4% for *L. mexicana*, and 6.1–11.9% for *L. infantum*, have been reported in Quintana Roo and the Yucatan peninsula [568–570]. A prevalence of 19% of visceral leishmaniosis has been reported also in dogs from Chiapas [571]. In cats, prevalences of infection with *L. mexicana*, *L. braziliensis* and *L. panamensis* at 10%, 11.6% and 22.1%, respectively, have been reported in Yucatan [569].

Trypanosomosis due to *T. cruzi* has been reported in dogs at seroprevalences of 8.1% in Jalisco, 7.6% in Campeche, between 4.5–42.8% in Chiapas, 20.0–21.3% in Quintana Roo, 21.0–24.5% in Tejupilco, 17.5% in Toluca and 9.8–34.0% in Yucatan [84, 572–579]. In cats, *T. cruzi* infection has been reported at a seroprevalence of 7.4–8.6% in the Yucatan Peninsula [580, 581].

#### Bacterial diseases

Anaplasmosis due to *A. phagocytophilum* was described in sick dogs from Oaxaca at a seroprevalence of 7.4% [582] and of 3% from Monterrey [583], but potential cross-reactivity in the used test system with *A. platys* antibodies should be borne in mind. *Anaplasma* sp. was also detected in a large countrywide screening at 0.61–16.4% seroprevalences all over the area, depending on the region [186]. A molecular prevalence of 31% for *Anaplasma* sp. was reported for Coahuila and Durango with 3% of the dogs confirmed as *A. platys* infection [584].

Lyme borreliosis in dogs due to *B. burgdorferi* (*s.l*.) is reported in variable range among different regions of Mexico. It was reported in 0.9% of dogs from Nuevo Leon (by PCR), in 16% from Monterrey, in 8.2% from Mexicali, and in 0.23% of dogs including 21 Federate Mexican states (by seroprevalence) [186, 187, 585, 586]. Seroprevalence in humans was 3.4% in Mexico City, 6.2% in northeastern regions, and 0.3% in a nationwide survey [587, 588], with the Northeast considered as a zone where Lyme disease is endemic [589].

Ehrlichiosis due to *E. canis* was reported at a seroprevalence ranging between 8.7–44.1% in dogs from Yucatan [590, 591], a seroprevalence of 74.3% in clinically suspected dogs from Sinaloa [592] and at a molecular prevalence of 45% in shelter dogs from Yucatan [593]. Similarly, a seroprevalence of 37% was reported in sick dogs from Oaxaca [582]. *Ehrlichia canis* was also detected in a large countrywide screening at seroprevalences of 2.4–51%, depending on the region [186].

Rickettsiosis due to *R. felis* or *R. rickettsii* has been reported in people, but not in dogs [594]. Nevertheless, *R. akari* has been reported in a dog from Yucatan, whereas *R. felis* has been reported at a prevalence of 20% in fleas collected from dogs also on the Yucatan Peninsula [595, 596] and *R. rickettsii* has been reported in *A. cajennense* collected from dogs [597].

### Nicaragua

#### Parasitic diseases

Babesiosis in form of *Babesia* spp. infection has been reported in dogs at a molecular prevalence of 26% (10/39), with four dogs being infected with *B. gibsoni* and six being infected with *B. vogeli* [598].

Dirofilariosis due to *D. immitis* has been described in two dogs from Managua [599], but autochthonous character of the two dogs is questionable. In a screening of 329 dogs a seroprevalence of 1.8% was detected. Additionally, in the same study in single dogs microfilariae were detected by microscopy and *D. immitis* infection was confirmed by PCR in two dogs [492].

Hepatozoonosis due to *H. canis* was detected at a molecular prevalence of 51% [598].

Leishmaniosis in different clinical scenarios and caused by different species has been reported in man [600, 601], but no prevalence data in dogs or cats could be found.

Trypanosomosis due to *T. cruzi* has been described in people in Nicaragua [602, 603]. No information about the prevalence of the pathogen is available in dogs or cats.

#### Bacterial diseases

Anaplasmosis in dogs due to *A. platys* infection has been reported at a molecular prevalence of 13% [598] and at a seroprevalence to *Anaplasma* spp. of 28.6% [492]. In the latter screening, *A. platys* and *A. phagocytophilum* infection could be confirmed on a molecular basis in 21.3% and 18.1% of seropositive dogs, respectively [492].

Lyme borreliosis could not be confirmed in a serosurvey of 329 dogs [492].

Ehrlichiosis in dogs has been found at a molecular prevalence of 56% [598] and at a seroprevalence of 63% for *E. canis* [604] and 62.9% for *Ehrlichia* spp. [492]. In the last study, 58.5% of all seropositive dogs were confirmed to be infected with *E. canis* by molecular methods [492].

Rickettsiosis due to *R. felis* has been reported at a molecular prevalence of 5% in dogs [598]. Additionally, *R. amblyommii* could be detected in *A. ovale* [605].

### Panama

#### Parasitic diseases

Leishmaniosis was detected in dogs by microscopic examination at prevalences ranging between 3.0–15.4% [606–609]. A seroprevalence as high as 47% in dogs has also been reported in endemic regions [610]. Trypanosomosis has been reported at an overall infection index of 16.2% in dogs, with a seroprevalence of 11.1% for *T. cruzi* and at an infection rate (determined by PCR and blood culture) of 5.1% for *T. rangeli* [611].

#### Bacterial diseases

Anaplasmosis due to *A. platys* has been detected at a molecular prevalence of 21.4% in dogs [612]. *Anaplasma phagocytophilum* has been identified in ticks collected from a cow [613]. Ehrlichiosis due to *E. canis* infection has been detected at a molecular prevalence of 64.2% in dogs [612]. Other *Ehrlichia* spp. have been detected in ticks from horses and cattle [477, 613]. Rickettsiosis has been reported in dogs at a seroprevalence of 55% for *R. amblyommii*, 20% for *R. rickettsii*, 5% for *R. bellii*, 25% for *R. rhipicephali*, 10% for *R. parkeri* and 15% for *R. felis* [614]. Similarly, *R. felis* and *R. amblyommii*/“*Candidatus* R. amblyommii” have been detected in fleas and ticks, respectively, from dogs and cats [477, 614–617].

### Paraguay

The information on vector-borne pathogens in Paraguay is extremely scarce or non-existent.

#### Parasitic diseases

Babesiosis in domestic dogs has been detected at an overall prevalence of 6% from 384 animals surveyed from Asunción, with *B. vogeli* being the most predominat piroplasmid species [618]. Dirofilariosis by *D. immitis* has been reported by necropsy in eight dogs of 200 street animals [619]. Leishmaniosis has been reported at seroprevalences ranging between 6.6–69.0% in dogs [620–622]. Trypanosomosis was detected in dogs at seroprevalences of 36.4% and 38% [623, 624] and in cats at 37.5% [624].

#### Bacterial diseases

Anaplasmosis has been detected in a larger population of dogs (*n* = 384) sampled from Asunción; *A. platys* was detected and identified at a molecular prevalence 10.67% [625]. Ehrlichiosis has been reported in the same population of dogs (*n* = 384) from Asunción with *E. canis* detected and identified at a molecular prevalence of 10.41% [625].

### Peru

#### Parasitic diseases

Dirofilariosis due to *D. immitis* has been reported at a seroprevalence of 4.4% in dogs from Lima [626, 627] and ranging between 0–12.8% seroprevalence in further studies from Lima [628–630].

Leishmaniosis has been reported in Peru at molecular prevalences ranging between 5.4–7.6% in asymptomatic and 18–45% in symptomatic dogs [631–634]. Prevalence was highly dependent on the detection method [631], as well as on the type of sample and the molecular target used for testing [632, 635].

Trypanosomosis due to *T. cruzi* infection in dogs has been reported in southern Peru at a seroprevalence of 12.3% [636], while in northern Peru seroprevalences ranged between 19.8–40.0% [637, 638].

#### Bacterial diseases

Anaplasmosis due to *A. phagocytophilum* infection has been reported in a single dog from Lima [639]. Caution should be enforced due to potential cross-reactivity of the used test with *A. platys*. *Anaplasma platys* infection as suggested by inclusion bodies in platelets, was identified in 29.2% of pet dogs from Lima, and a prevalence of 1.4% for *A. platys* was detected by molecular methods in the same dog population [640].

Bartonellosis due to infection with *B. rochalimae* or *B. vinsonii berkhoffii* was detected by molecular methods in 10% of asymptomatic dogs [641]. The same survey also showed a seroprevalence of 62% for *B. rochalimae* and of 40% for *B. vinsonii berkhoffii*. Infection with *Bartonella* species in cats has been reported [642], but no prevalence values are available.

Lyme borreliosis has been reported in people in Peru [643, 644]. Furthermore, potential vectors have been detected [643], but information is scarce. Seropositivity has been reported in two dogs from Lima one of which was suspected to be of autochthonous character [639].

Ehrlichiosis has been reported in dogs [626, 645] as well as in humans, here in form of seropositivity to *E. canis* and *E. chaffeensis* [222, 645, 646]. A survey of a small cohort of dogs showed a molecular prevalence of 44% for *E. canis* [645] and a seroprevalence of 16.5% for *E. canis* in a population of 140 dogs [626].

Rickettsiosis in Peru has been reported in people and vectors [647]. A seroprevalence of 59.2% in dogs and of 7.7% in cats has been reported for spotted fever group rickettsiae [647]. Similarly, *R. felis* and *R. parkeri* have been detected in fleas and ticks from domestic animals [648].

### Puerto Rico

The information on vector-borne pathogens in Puerto Rico is scarce.

#### Parasitic diseases

Dirofilariosis due to *D. immitis* in dogs has been detected at a seroprevalence of 19% in 123 dogs tested from Guaynabo and Ponce regions [649] and of 6.7% in 1,723 dogs with massively varying prevalences (up to 20.4%) in the different tested locations on the island using Knottʼs test [650]. A seroepidemiological study in humans revealed 2.66% *D. immitis* seropositives [651].

#### Bacterial diseases

Anaplasmosis due to *A. phagocytophilum*, showed a seroprevalence of 30.9% for 123 dogs from Ponce, Guaynabo and Vieques Island [649], but caution should be considered due to cross-reactivity with *A. platys* antibodies in the used test system. Lyme borreliosis in dogs has not been detected by a serological survey in Guaynabo, Ponce or Vieques Island [649]. Ehrlichiosis due to *E. canis* has been detected at a seroprevalence of 45.5% in dogs [649].

### Suriname

The information on vector-borne pathogens in Suriname is extremely scarce or non-existent.

#### Parasitic diseases

Dirofilariosis in dogs by *D. immitis* infection has been reported in old dissection studies [652–655] and by Panday et al. [656] detecting 26% of positive dogs using modified Knottʼs test and 5.7% of seropositive dogs using IFA test. Leishmaniosis in form of human cutaneous leishmaniosis is endemic in the hinterland [657–661] and has been detected in a population of 47 dogs with a seroprevalence of 4.3% [662, 663]. Trypanosomosis suspected to be caused by *T. evansi* has been reported in four single cases in hunting dogs [664] and due to *T. cruzi* is reported in people [665].

### Uruguay

#### Parasitic diseases

Leishmaniosis has recently been reported in 11/45 dogs by serology in Salto, Uruguay. Typing revealed *L. infantum* as corresponding pathogen. Additionally, *Leishmania* DNA was also detected in sand flies [666]. Trypanosomosis has been described in people in Uruguay [667–670], but no reports or prevalence data are available for dogs or cats.

#### Bacterial diseases

Anaplasmosis due to *A. platys* infection has been reported in 4.2% of dogs surveyed in northwestern Uruguay [671]. Bartonellosis was not reported in dogs or cats, but has been described in children [672, 673]. Lyme borreliosis was not described in people, dogs or cats. Nevertheless, *B. burgdorferi* (*s.l*.) genospecies have been detected in *Ixodes pararicinus* (*I. ricinus* complex group) ticks in the region [182]. Rickettsiosis due to seroreactivitiy against antigens of *R. felis*, *R. parkeri* and *R. rhipicephali* has been described in dogs at an overall seroprevalence of 20.3% [674]. From that study, it is estimated that at least 14% of dogs were seropositive for *R. parkeri*, or a *R. parkeri*-like organism. *Rickettsia parkeri* and *R. felis* have furthermore been detected in ticks and/or fleas [674–678], and there have been reports on *R. conorii* infections in humans [679, 680], but with some debate on cross-reactivity [678].

### Venezuela

#### Parasitic diseases

Babesiosis due to *B. vogeli* has been reported at a molecular prevalence of 2.2% in dogs [681].

Dirofilariosis has been reported using modified Knottʼs test at a prevalence of 15.8% in dogs from Sucre [682] and, using ELISA, at a prevalence of 13% in Barquisimeto [683] and of 44.9% in Maracaibo [684]. D’Alessandro [685] reported an overall prevalence of 28.9% in dogs from Aragua using microscopic blood examination; the author detected a higher prevalence in hunting dogs (58.5%) compared to shelter or owned dogs (11.7%). Furthermore, there are also single feline case reports published for Venezuela [686–688].

*Hepatozoon* infection in dogs due to *H. canis* has been reported at a prevalence of 44.8% [681].

Leishmaniosis in dogs has been reported at prevalences ranging between 3–57%, depending on the region, the year and the type of test [323, 689–692]. On Margarita Island, seroprevalences of 21.0–33.1% have also been reported for dogs [693].

Trypanosomosis has been reported in dogs at seroprevalences ranging between 6.4–67.6% [694–698].

#### Bacterial diseases

Anaplasmosis in dogs due to *A. platys* has been reported [699, 700], and in one study even a prevalence of 16% by PCR was documented [701]. Lyme borreliosis has been described in humans [702, 703], but no reports on dogs or cats are available. Ehrlichiosis due to *E. canis* infection has been reported at a molecular prevalence of 31% in dogs [704]. A co-infection in a dog with *E. canis* and *E. chaffeensis* has been also reported [705].

## Summary and priorities in companion vector-borne disease management

As illustrated by the prevalence data presented in this review, vector-borne pathogens are ubiquitous in LATAM, and represent a challenge for animal and, due to the zoonotic character of several of them, public health systems in both, urban and rural environments.

Unfortunately, diagnosis of VBDs as well as the system of VBD surveillance, reporting, prevention and control in the region is relatively weak, very limited, and in most cases inexistent.

During the last ten years, significant improvements in vector control and surveillance, clinical diagnosis, and medical practices have been achieved in the area of VBDs globally, but this seems not be the case for several areas in LATAM. Regrettably, LATAM is characterized by an expanding human population with marked social, cultural and economic inequalities. Several factors have created conditions for the emergence and persistence of previously unrecognized vector-borne and zoonotic diseases in most of the countries of the region [11, 38, 706], such as drastic changes in economic development and land use; poor waste disposal management practices (conducing to an uncontrolled growth of feral dog and cat populations); absence of responsible pet ownership; lack of awareness of animal welfare and disease prevention; restricted economic constrains to proper veterinary care; and extremely limited access to technological advances in diagnostic tools. Under these circumstances, it is clear that one of the most important steps towards control of CVBDs is prevention. In this context, companion animals, often having higher exposure and risk factors to VBDs than humans, could play a valuable role in minimizing the zoonotic potential of CVBDs by controlling this reservoir through proper prevention.

Prevention of infection should be based on actions aimed at averting infection in three main areas: vector control through use of repellent ectoparasiticides/insecticides and through environmental control (control of water accumulation, waste management, insecticidal treatment, mosquito screens etc.), vaccination, where applicable, and behavioral prophylaxis (cleaning of animals’ residues, avoidance of daily phases with high vector activity like e.g. twilight, no abandonment of pets etc.).

Several previously unrecognized or overshadowed vector-borne pathogens that affect companion animals are present in LATAM. Most, if not all of the diseases presented here are zoonotic, which not only represents a concrete risk for pet animals, but also for people. Unfortunately, the information to the veterinary, public and medical community is either very scarce, limited, inexistent or not accessed and due to non-awareness in the people concerned.

In order to address the challenges that CVBDs impose to the region, some of the following priorities should be considered:

- Availability of affordable diagnostic techniques with solid interpretation and easy access to diagnostic reference laboratories in order to maintain consistent methodologies and updated diagnostic techniques.
- Easy access to formal (i.e. scientific and medical journals) and informal (i.e. conference and meeting proceedings, white papers, etc.) information regarding occurrence of VBDs, new or improved diagnostic tools, clinical findings, treatment protocols, and options of prevention aimed at veterinarians and medical professionals.
- Creation of cooperative extension services and outreach programs fostering the collaboration between veterinarians, physicians, scientists, health workers, social workers, educators and farm communities.
- Development of impactful educational programs aiming at pet owners, farmers, and the general public regarding responsible pet ownership, vector control and VBD prophylaxis.
- Development of VBD surveillance network systems in collaboration with state and local health departments.

For veterinarians these priorities can be expanded into concrete actions as summarized also in Baneth et al. in a similar way [707]:

- Forget about exotic diseases as any disease can occur in the practice.
- Stay informed with up-to-date research data *via* diverse channels.
- Prevent transmission as best approach to CVBD management.
- Include fleas onto the list of potential vectors.
- Consider non-vectorial transmission in the case of leishmaniosis, *Bartonella* and hemotropic mycoplasmas.
- Check for the patients’ travel schedule.
- Inform yourself on proper diagnostic methods.
- Consider treatment not necessarily as end of an infection.
- Inform and keep in touch with your clients.
- Alert public health authorities where appropriate.

VBDs are among the most complex of all infectious diseases and may pose a challenge to mitigate, control and prevent. A true One Health approach is required to respond to the current challenges presented by these diseases in both humans and animals. In LATAM, the actions towards mitigating the impact that CVBDs impose to both animal welfare and public health are intimately tied to the economic, social, and political values of the people in the region.

An interdisciplinary cooperation between professionals in human and animal medicine, scientists, ecologists and sociologists, a truly One Health approach, should be encouraged to ensure that surveillance is linked to actions. The creation of extension services at community levels providing culturally and economical acceptable veterinary services, including access to information, prevention, diagnosis and treatment to underserved regions, will be the key to minimize the impact of these diseases in the region. For the start, as preventing is always preferable to curing, the presumably easiest action to be taken here is a strong call for year-round prevention of pets with suitable and highly effective ectoparasiticides and microfilaricides (and where applicable also vaccines).

## Conclusions

VBDs in companion animals possess a wide distribution in LATAM. But in contrast to this wide distribution, data availability and accessibility on the occurrence of the different diseases are very different for the individual countries of LATAM and often scarce. Some countries, e.g. Argentina and Brazil, possess profound data availability, whereas especially in some of the smaller ones international accessible data is missing. Generally, none of the examined LATAM countries is completely free f the listed pathogens in companion animals. The fact that some of the discussed diseases and pathogens possess zoonotic character demands for a strong call for disease prevention in companion animals by repellent ectoparasiticidal/insecticidal control, environmental control, vaccination, where applicable, and behavioral prophylaxis. Behavioral priorities especially also for veterinarians and a One Health approach are needed for the region.

## Abbreviations

AHS: American Heartworm Society
CVBD: companion vector-borne disease
LATAM: Latin America
LB: Lyme borreliosis
TroCCAP: Tropical Council of Companion Animal Parasites
VBD: vector-borne disease
